# Self-explaining roads: What does visual cognition tell us about designing safer roads?

**DOI:** 10.1186/s41235-021-00281-6

**Published:** 2021-03-04

**Authors:** Jan Theeuwes

**Affiliations:** 1 Department of Experimental and Applied Psychology, Vrije Universiteit Amsterdam, Van der Boechorststraat 7, 1081 BT Amsterdam, The Netherlands; 2 Institute Brain and Behavior Amsterdam (iBBA), Amsterdam, The Netherlands

## Abstract

In 1995, Theeuwes and Godthelp published a paper called “self-explaining roads,” in which they argued for the development of a new concept for approaching safe road design. Since this publication, self-explaining roads (SER) became one of the leading principles in road design worldwide. The underlying notion is that roads should be designed in such a way that road users immediately know how to behave and what to expect on these roads. In other words, the environment should be designed such that it elicits adequate and safe behavior. The present paper describes in detail the theoretical basis for the idea of SER and explains why this has such a large effect on human behavior. It is argued that the notion is firmly rooted in the theoretical framework of statistical learning, subjective road categorization and the associated expectations. The paper illustrates some successful implementation and describes recent developments worldwide.

## Significance statement

In 1995, the idea was put forward that a traffic environment *should elicit safe behavior simply by its design.* This idea was dubbed *self-explaining roads* (SER), and over the course of 25 years, this approach became the leading principle of road design worldwide. The current paper describes the psychological principles underlying the concept of SER and describes current trends and developments.

## Introduction

In a paper published in 1995, Theeuwes and Godthelp were the first to introduce the concept of *self-explaining roads* (SER) as the leading principle of road design (Theeuwes and Godthelp 1995; Theeuwes 1998). The basic notion of a self-explaining road is a “*traffic environment which elicits safe behavior simply by its design*” (Theeuwes and Godthelp 1995, p. 217). The underlying idea is that the design and layout of the road environment elicits automatically the behavior that is appropriate for that type of road. In other words, the road nudges the right behavior without the need for much enforcement or education.

Since its publication, the notion of SER has gained a lot of momentum and is now considered the main design principle for road authorities and departments of transportation worldwide. In many countries across the world, roads were redesigned and adapted such that the road was adapted consistent with the SER principles. The EU Mobility and Transport committee also adopted this principle (see web) and has funded several research projects focusing on this issue. In 2010, the World Health Organization (WHO) and the United Nations (UN) explicitly mentioned the need for “*promoting the safe system approach and the role of self-explaining and forgiving road infrastructure*” proclaiming the decade of action for road safety.

In 2010, Charman et al. (2010) published an extensive literature review regarding self-explaining roads and the various approaches to the concept. This review was part of a larger SPACE project funded by the 6^th^ framework of European Commission as part of the European Research Area Network ROAD. Charman et al. (2010) concluded that “*the self-explaining road message fell on fertile ground, and within a decade the terms self-explaining roads concept, self-explaining road principles, and even self-explaining road philosophy were in wide-spread use, not just in Europe but across the globe, and often in situations far-removed from those envisaged by the original authors*” (Charman et al. 2010, p. 10).

When initially publishing this paper in 1995, we could never have foreseen the impact of our ideas on the way engineers, scientists and policy makers approach road safety. In the original publication, we did not provide a solid theoretical basis for the principles underlying SER. The goal of the present paper is to provide this theoretical basis. It is argued that visual selection during driving is very much determined by what has been labeled by basic visual cognition theories as “selection history” (Awh et al. 2012; Theeuwes 2019). According to this idea, our past experiences of paying attention to certain objects or events and not to others have a strong and enduring effect on what will be attended when we encounter similar contexts again. We argue that the effect of past driving experiences will bias visual selection in an implicit and automatic way, explaining why it is so important to design roads which are consistent with these automatic biases. We highlight the role of statistical learning during driving, subjective road categorization and the associated expectations. The paper illustrates some successful implementations and describes recent developments worldwide.

## Self-explaining roads: the underlying theory

### Background

It is generally agreed that most traffic accidents are related to human error (e.g., Treat et al. 1977; NHTSA 2016). Because educating road users and mass media campaigns directed towards changing road users behavior only have limited effects (see Wakefield et al. 2010), it is crucial that the road environment elicits adequate behavior and minimizes human errors simply by its design. Driving a vehicle (especially among those who are experienced drivers) involves hardly any conscious control and basically consists of several skill-based components that are fully automatized (Wickens and Horrey 2009). It has been argued that behavior can be triggered automatically by features in the environment (Bargh and Ferguson 2000), suggesting that drivers may display behavior that is inconsistent with their explicit goals. For example, drivers coincidently following a familiar route to get to another new destination may find themselves following the familiar route too far. Also, in a driving simulator study, it was shown that after driving the same route 24 times over four days participants failed to notice that an important road sign had changed (Martens and Fox 2007).

Selecting relevant information and avoiding distracting information is crucial during driving. It has been estimated that over 90% of the information that a driver has to process is visual (Hills 1980; Sivak 1996; Spence and Ho 2015). It is also known that deficiencies in visual attention are responsible for a large proportion of road traffic accidents (Charlton and Starkey 2013; Sabey and Taylor 1980). Research on hazard perception has shown that drivers with good hazard perception skills are less involved in accidents than those drivers with low hazard perception, for example novice drivers (McKenna and Crick 1991; Scialfa et al. 2011). Critically, accident data have shown that in many cases, drivers that are involved in automobile crashes do not act too late but do not act at all to avoid the collision (Guo et al. 2010), suggesting that they simply did not attend to the event that ultimately resulted in a crash. Detection of potential hazards is particular difficult when the road environment is complex and unusual and the cognitive load is relatively high, for example when drivers are relatively inexperienced or drive in a foreign city (Kahana-Levy et al. 2019; Underwood et al. 2002).

The crucial point is that in many cases human error plays a large role in road crashes. For example, according to a study from 2016 of the National Highway Transportation Safety Administration (NHTSA 2016), in the USA somewhere between 94 and 96% of all car accidents are caused by human error. Instead of blaming the driver for making these errors, in many cases, road crashes are caused by design-induced errors (Dumbaugh et al. 2020). This indicates that the road design may have been so confusing, inconsistent and violating the expectancies of road users that errors are likely to occur even when road users actively try to prevent them. Making roads “self-explaining” focuses on a road-user-adapted design using road elements such as signs, markings, geometry, road surface, lighting, road surface, traffic and speed management, to prevent errors from occurring. In addition to preventing errors from occurring, another important aspect of a safe infrastructure is to make roads more “forgiving” implying that if an error is made, its consequences are minimized. In other words, the design of the road should forgive the driver for making errors by reducing the severity of accidents (Nitsche et al. 2010).

From a theoretical point of view, it is assumed that selecting information from the environment is the result of the interaction between intentions and the goals of the driver (current selection goals) and the physical properties of the visual environment (saliency of the objects). Many basic models of attentional control have described selection as a result of this interaction between what are referred to as “bottom-up” and “top-down” processes (Corbetta and Shulman 2002; Itti and Koch 2001; Theeuwes 2010) sometimes referred as stimulus-driven and goal-driven selection (Egeth and Yantis 1997; Ludwig and Gilchrist 2002), or automatic and non-automatic control (Shiffrin and Schneider 1977). To give an example from the driving task: In case a driver wonders whether he is allowed to turn right on a given street, he/she may actively search in a top-down fashion for information (signs, road markings) telling him/her whether this is allowed, while at the same time his/her attention may be captured in a bottom-up way, by a street worker wearing an orange fluorescent jacket.

### Statistical learning and visual selection

**Fig. 1:**
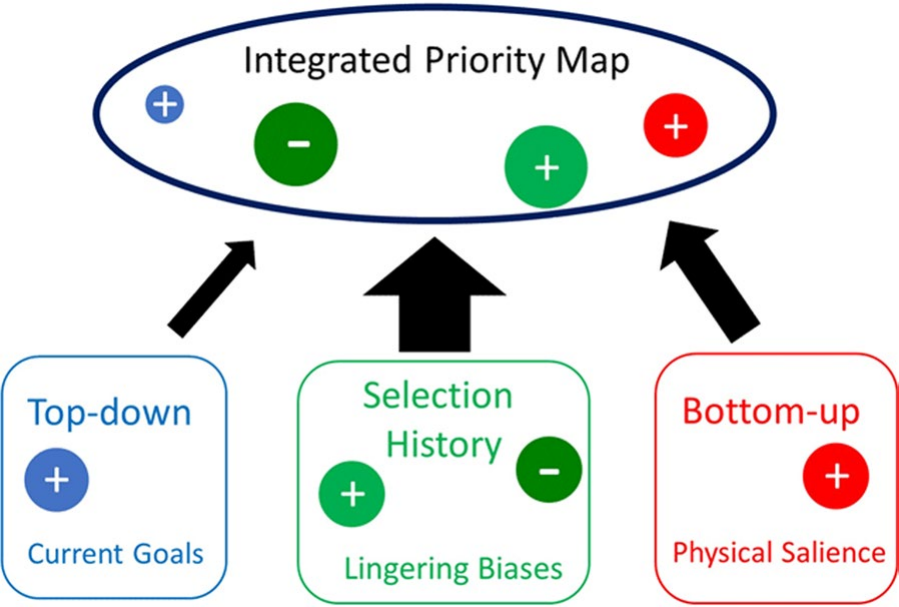
A schematic representation of how a priority map that integrates three sources of selection biases: the observer’s current selection goals, selection history, and the physical salience of the items competing for attention (adapted from Theeuwes 2019). The smaller blue plus sign as part of the priority map indicates that the role of top-down selection is rather limited (Theeuwes 2018; but see Wolfe 2021)

Even though it is generally agreed that top-down and bottom-up factors are important in visual selection, recently it was pointed out that that this classic theoretical dichotomy no longer holds as in many cases attentional selection can neither be explained by current selection goals nor by the physical salience of potential targets (Awh et al. 2012; Theeuwes 2018, 2019). Awh et al. (2012) suggested a third category which they labeled “*selection history*” to stress that the history of attentional deployments can elicit lingering and enduring selection biases, unrelated to top-down goals or the physical salience of items. The history of these previous selection episodes enables the cognitive system to extract regularities from the environment. Statistical learning (SL) is the mechanism underlying the ability to extract the distributional properties from sensory input across time and space (Frost et al. 2015). These three factors (see Fig. 1) all feed into an integrated priority map which represents a conceptual framework accounting for selection priority. It is assumed that competition between the input from current goals, physical salience and selection history determines, in a winner-take-all fashion, the object that ultimately will be selected.

Relative to top-down and bottom-up factors affecting visual selection, recently it was recognized that “selection history” may represent a much more important factor affecting visual selection than previously assumed (Failing and Theeuwes 2018; Theeuwes 2019). Specifically, the cognitive machinery is tuned to the structured properties of the environment, such that given a particular context, our senses "expect" particular input at a particular time and particular place. SL is one of the most fundamental abilities of any living organism. With respect to the visual domain, so-called contextual cueing studies have shown that observers can learn that particular stimuli co-occurred frequently and occurred often in particular locations within the display (Chun and Jiang 1998, 1999). This type of learning is considered to be largely unconscious, incidental or implicit, indicating that learning occurs automatically without instruction (Duncan and Theeuwes 2020) and without the observers necessarily knowing that they selectively attend specific patterns. SL is thus often considered to be the passive absorption of regularities in the environment.

In a labortory environment, specific regularities in the environment are introduced to examine the extent to which these regularities are learned and, how in turn, they bias attentional selection. For example, a pattern in which targets appear more frequently in one region of visual space is implicitly learned over repeated exposures (Ferrante et al. 2018; Geng and Behrmann 2005; see Jiang 2018 for a review) showing that participants find these targets faster and more efficiently. Similarly, participants also can learn to avoid locations that are more likely to contain distracting information (Wang and Theeuwes 2018a, b, c). Yet, much more important than regularities introduced in the laboratory are the regularities learned during a lifetime. For example, the classic work of Biederman (1972; Biederman et al. 1982) demonstrated that in each environment, we expect that particular objects often co-occur and we expect that within those environments, objects typically are found at specific locations. For example, a coffeemaker, a pan and a knife are likely to be found in a kitchen, and within that kitchen these objects are often positioned in a particular location (on the countertop, probably not on the floor). Also, objects violating the regularities learned over a life time are more difficult to find, for example when these objects are presented at inconsistent locations within a scene. Likewise, objects that appear in a scene context that is inconsistent are more difficult to identify (e.g., Biederman et al. 1982). Recently, Võ and Wolfe (2013) refined this work and made a distinction between semantic and syntactic scene-object relationships referring to the type of objects and where these objects are likely to be found within a scene.

### The road environment and SER

The claim here is that statistical learning plays a crucial role when people interact with the road environment. Critically, within any given road environment, experienced drivers have learned to expect particular relevant objects to appear in particular locations similar to what basic studies have shown with respect of expecting particular objects to appear at particular locations within, for example, a kitchen or a bedroom scene (Biederman 1972; Võ and Wolfe 2013). Through experience, drivers have learned to extract the regularities from the road environment. This implies that they have learned to expect particular objects to co-occur and have learned to expect to find particular critical objects and potential hazards in the appropriate locations and expected moments in time within the scene. Because road users have to rely on this learning experience, selection from the road environment is efficient, adequate and swift. If a road environment is well designed (i.e., these expectations induced are not violated), one speaks of a road which is *self-explaining* as the design and layout of the road environment elicits automatically the appropriate behavior for that type of road.

Evidence that road users have learned to expect particular object to appear at particular locations within a given road scene comes from a study by Theeuwes and Hagenzieker (1993; see also Theeuwes 1996). In Theeuwes and Hagenzieker’s experiment, on each trial participants (experienced drivers) were required to search for a particular object within the road-scene and decide whether this object was present or not. For example, participants had to search for a bicyclist, a traffic sign or a pedestrian within a scene, and these objects were placed at the appropriate location within the scene (e.g., a bicyclist on a bike path, a traffic sign on the right side of the road, and pedestrian at a zebra crossing) or at an inappropriate location (e.g., a bicyclist not on the bike path but on the expressway, a traffic sign on the left side of the road and a pedestrian crossing not at the zebra crossing but in the middle of the street). The results showed that participants were faster and more accurate finding objects that were positioned in the appropriate location than objects in the wrong location. Often participants responded “object not present” while in fact the target object was present but at an unexpected location.

In a follow-up study, Theeuwes (1996) measured eye movements while participants viewed video clips of intersection approaches. Participants (experienced drivers) were required to search for a blue traffic sign which could, given the layout of the scene, be located at an appropriate location or an inappropriate location within this scene. The results revealed the importance of the learned regularities: Eye movements were first directed to those locations where target objects were likely to be found (e.g., on the right side of the road), before the eyes were directed to the less likely locations.

A field study by Räsänen and Summala (2000) provides direct empirical evidence for the role of learned regularities in driving. In this study which was conducted in Finland, Sweden and Denmark, car drivers, who did not know they participated in an experiment, approached a roundabout while remote camera’s measured the driver’s head movements. A test (stunt) cyclist who was part of the experimental setup created well-controlled conflict situations with the approaching car. The study showed that drivers entering roundabouts tended to direct their attention (as defined as the direction of head movements) mainly to the left side of the visual field. Critically, this direction of attention did not dependent on whether traffic was coming from the left, but mainly based on learned expectations (traffic is typically coming from the left on these roundabouts). One important finding was, however, that there is a price to pay for search behavior that was guided by learned regularities: In many instances, a cyclist coming from the right did not generate a head movement suggesting that drivers failed to detect the cyclist when it approached the roundabout from the right. Even though the authors recognized that the absence of head movements in the direction of the cyclist does not necessarily imply that they did not detect the cyclist, additional evidence showed that those drivers that did not look to the right typically failed to yield to the cyclist. This latter finding suggests that indeed the car driver failed to detect the cyclist from an unexpected direction (in this case from the right). Note that the study was conducted during daytime in the summer, and therefore, it is likely that the cyclist was clearly visible (see also Theeuwes 2000 for a discussion).

The general notion here is that a particular road environment elicits particular expectations learned during driving which in turn biases search behavior towards those portions of the visual field where relevant information (including potential hazards) is expected. It should be realized that these biases due to learned regularities will be particularly strong in conditions of high workload, i.e., driving in busy traffic in urban environments, or under reduced sight conditions. In those circumstances, drivers need to rely on fast and automatic extraction of the relevant information from the environment. Our notion is that under high load conditions, potentially relevant objects at unexpected locations are not seen too late but, in most cases, not seen at all, i.e., road users may simply overlook the presence of unexpected relevant objects or other road users. As noted, accident data confirm this notion as drivers often involved in automobile crashes do not act too late but do not act at all to avoid the collision. Guo et al. (2010) analyzed the data of the 100-Car Naturalistic Driving Study (2006) and showed that about 34% of crashed drivers did not react at all before the crash occurred.

Given these considerations, it is clear that extremely dangerous situations can occur when the design of the traffic environment induces certain expectations regarding the spatial arrangement of objects, potential hazards and other relevant road users in that road scene, which are not correct. In other words, the need for roads in which the design is consistent with the behavior required and consistent with the expectations that these roads induce, aka self-explaining roads, is quite high. We argue that dependent on the road environment, drivers have learned to expect particular road elements (signs, lights, markings) and road users (cars, bikes, pedestrians) to appear at particular locations within specific road categories (highway, rural road, city roads) (see Theeuwes et al. 2012; Theeuwes and Godthelp 1995).

One of the challenges is to determine what expectations particular road environments elicit. Take as an example a kitchen scene. People may have learned where things are in their own kitchen, but they also have a pretty good idea where things are when they walk into a kitchen they have never been before. What they have learned regarding the regularities in their own kitchen generalizes to basically all kitchens, or at least to kitchens which have basically the same setup (i.e., this may be different for American versus European kitchens). So the idea is that once an environment has been classified as a kitchen all biases are automatically retrieved determining how we interact with a kitchen environment. With respect to the road environment, the question is how drivers categorize a given road environment and whether this categorization is in fact correct given the behavior that is required on that road. For example, if a wide 4-lane road with smooth asphalt and clear markings is categorized as a motorway/freeway while it is in fact a 80 km/h road, it is difficult for drivers to keep to an appropriate speed because the road is automatically categorized as a motorway in which high speeds are expected.

The idea that people categorize objects and environments is based on the general notion that people try to structure their world. Structuring the world allows us to generalize across different objects and environments. For example, because we generalize across objects and environments, we are able to find a knife in a kitchen, even if we have never been in this particular kitchen. With respect to objects, it is known that people classify objects as belonging to a particular category (e.g., Rosch 1978). Through experience with particular objects, an internal representation is developed, which contains the typical characteristics of a particular category (Huth et al. 2012).). The same holds for the categorization of environments (e.g., Russell and Ward 1982). People not only distinguish environments on the basis of physical characteristics, but also on the basis of goals or the behaviors that take place in that environment (Russell and Ward 1982). It has been suggested that a "graded structure" is present within environmental categories; i.e., one environment is a better example than another for a particular category. If we take this to the road environment, classifying a road as a motorway will immediately instantiated particular expectations regarding the physical characteristics of the road (in the Netherlands: overhead signs, white road markings, emergency lane, etc.), the behavior of other road users (fast driving, overtaking, no pedestrians) as well as expectations regarding what behavior is allowed.

It is generally agreed that there are principles for categorization: *cognitive economy* and *perceived world structure* (Rosch 1978). Cognitive economy refers to the function of categorization in that it provides maximum information with the least cognitive effort. Perceived world structure refers to the structure of the information and asserts that the perceived world comes as structured information rather than as arbitrary or unpredictable attributes. Thus, maximum information with least cognitive effort is achieved when categories map onto the perceived world structure as closely as possible. When we apply the principle of *cognitive economy* to the road environment, it is reasonable to assume that road users try to reduce the large number of roads that exist in the "real" world to a few behaviorally and cognitively relevant road categories. It is only useful to differentiate between road categories where a difference between these categories is behaviorally relevant, for example the difference between roads where one can expect pedestrians to cross the road versus roads that do not have pedestrians at all. We assume that when a particular road is classified as being part of a certain road category it means that this road is similar to all other road environments within that category and different from roads outside that category. When we apply the principle of *perceived world structure* to the road environment, it implies that road users perceive the environment consisting of a set of attributes which are highly correlated. Clearly, a road environment is not made up of a randomly picked set of attributes; instead the attributes co-occur and serve a function. Thus, through statistical learning, road users develop a perceived world that contains attributes that are likely to occur in combination. In other words, if one sees a four lane motorway/freeway, one expects (at least in the Netherlands) an emergency lane, overhead signs, no crossing traffic, no traffic lights, wide road markings and fast traffic that moves in the same direction.

**Fig. 2:**
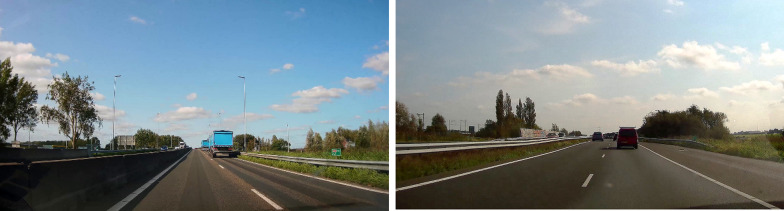
Examples of roads in the Netherlands that are difficult to categorize. On the left is a picture of a motorway/freeway (A44) without crossing traffic, with no slow moving vehicles, and expected speeds of 100 km/h (daytime) and 120 km/h (nighttime); while on the right is an expressway (N11) where traffic lights, traffic that is crossing (including bicyclists) and stopped vehicles can be expected while the maximum speed is 100 km/h. Because the roads look similar people categorize them as being the same, and will behave the same even though officially these are different types of roads (see Theeuwes and Diks 1995)

Research investigating how people subjectively categorize road environments showed that the subjective categorization (the way people categorize and perceived the roads in their "heads") may not necessary match the official road categorization (Theeuwes and Diks 1995). See Fig. 2 as an example. Theeuwes and Diks (1995) showed that participants may categorize roads that look similar (see Fig. 2) as belonging to the same road category while in reality the road on the left is a motorway/freeway without crossing traffic (the A44 in Netherlands), no slow moving vehicles, and a driving speed of 100 or 120 km/h; while the road on the right (the N11) is an expressway (100 km/h) where traffic lights, traffic that is crossing (including bicyclists) and stopped vehicles can be expected. This study showed that when roads look similar, they will be categorized as the same, and therefore road users will behave the same even though officially these roads are different (and therefore different behavior (and expectations) is required on these roads).

### Hypothetical model

**Fig. 3:**
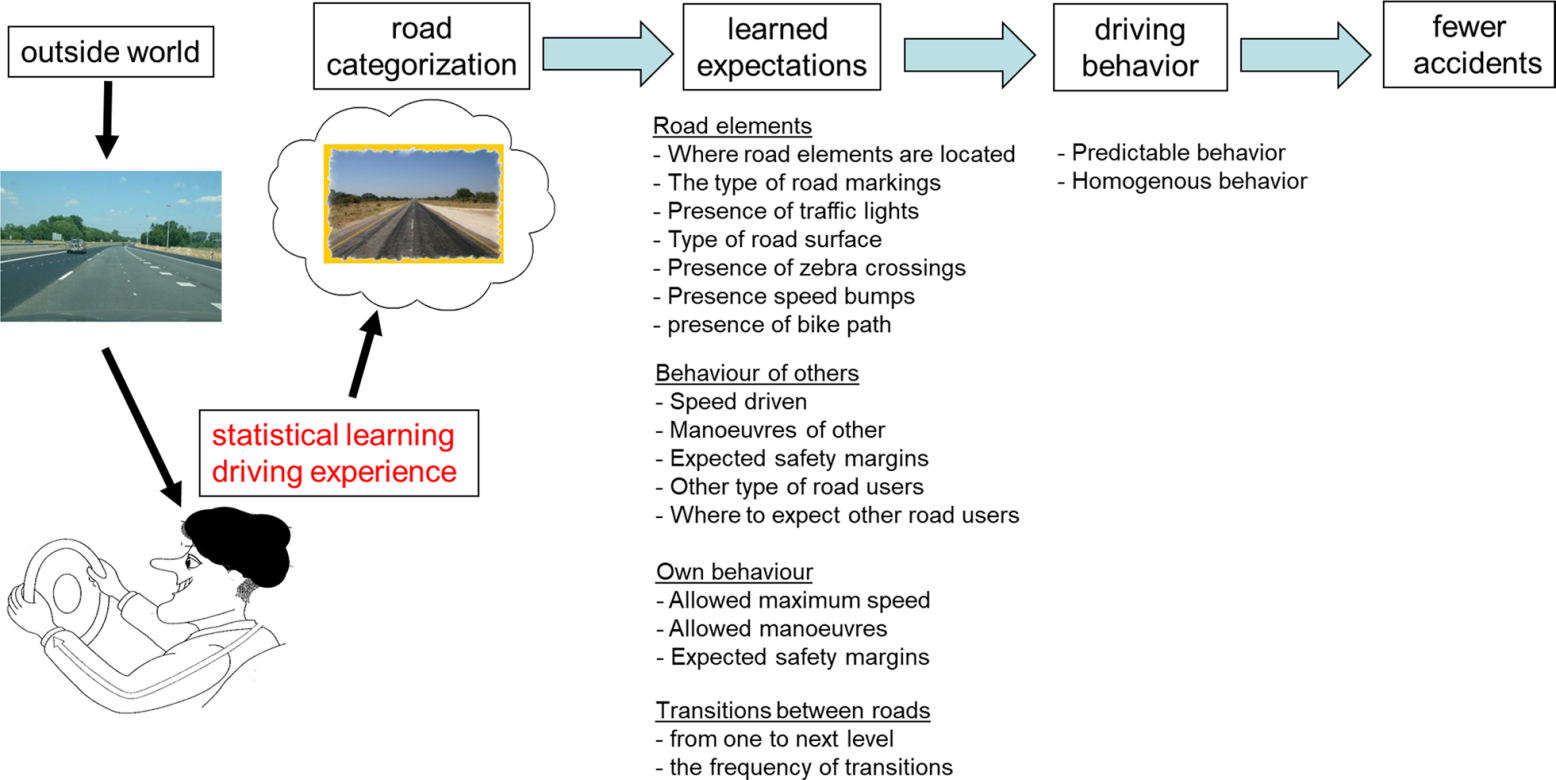
A hypothetical model explaining the relationship between statistical learning and subjective road categorization and how this affects road behaviour (adapted from Theeuwes et al. 2012)

We assume that through statistical learning, drivers learn to extract regularities from the road environment into a few behaviorally and cognitively relevant road categories. Once a road is classified, it will induce particular expectations, regarding the road elements that can be encountered (e.g., no zebra crossing or traffic lights on motorways/freeways), the other type of road users that are likely to be present on that road (for example there are not bicyclists or pedestrians on motorways/freeways), the likely behavior of other road users (e.g., will I be overtaken, nobody drives faster than 50 km/h), one’s own behavior (e.g., maximum speed), and transitions (e.g., there cannot be a direct transition from a motorway to a city road). Figure 3 presents a hypothetical model of how road design is connected to driving behavior and accident rate.

The hypothetical model can explain why adequate road design can ultimately result in fewer accidents. When a road is adequately classified, drivers will rely on their experience to scan the road environment and anticipate hazardous events. The flipside is that extremely dangerous situations can occur when drivers categorize a particular road environment inadequately. For example, if a driver categorizes a road as being a motorway (because it looks like a motorway) while in fact it is a provincial expressway, he/she would not expect that there could be crossing traffic or a slow moving farm vehicle. In these circumstances, accidents are prone to happen. To ensure that road users do not make these types of errors, the design of roads should be consistent with these learned expectations (Theeuwes and Godthelp 1995).

The model also makes clear that only when roads are clearly recognizable, all road users will all categorize similarly and only then will this result in homogenous expectations and behavior. If the physical appearance of the road environment is very heterogeneous (for example expressways in and outside the build-up area with different layouts and driving speeds), drivers are unable to extract and learn consistencies, resulting in inconsistent categorization and thereby heterogeneous road behavior. Research has shown that heterogeneous road behavior is associated with higher accident rates (Wegman 1995).

### Implications

#### Which roads are self-explaining; which roads are not

Of all road types, motorways/freeways are probably most self-explaining. These roads are designed with a purpose in mind. Because these roads are designed to allow a fast connection between important cities, there usually have smooth asphalt that allows fast driving, there is no opposing or crossing traffic, there are no traffic lights, there is usually an emergency lane, there are guard rails to separate opposing traffic, curves are never sharp, there is a lot of preview, there are no slow moving vehicles nor bikes, and usually, they have wide lanes. The design of the motorway fits perfectly with its function, and these roads induce expectations that are fitting. Only when expectations are violated (e.g., a sudden traffic jam; a sudden narrowing of the road; a pedestrian walking along the motorway), accidents on these types of roads are bound to happen. When the road unexpectedly narrows, often times the driving speed is too high to prevent an accident. Objects that do not belong on these roads (e.g., pedestrians) are often simply overlooked, and often this will result in a fatal crash with the pedestrian.

**Fig. 4:**
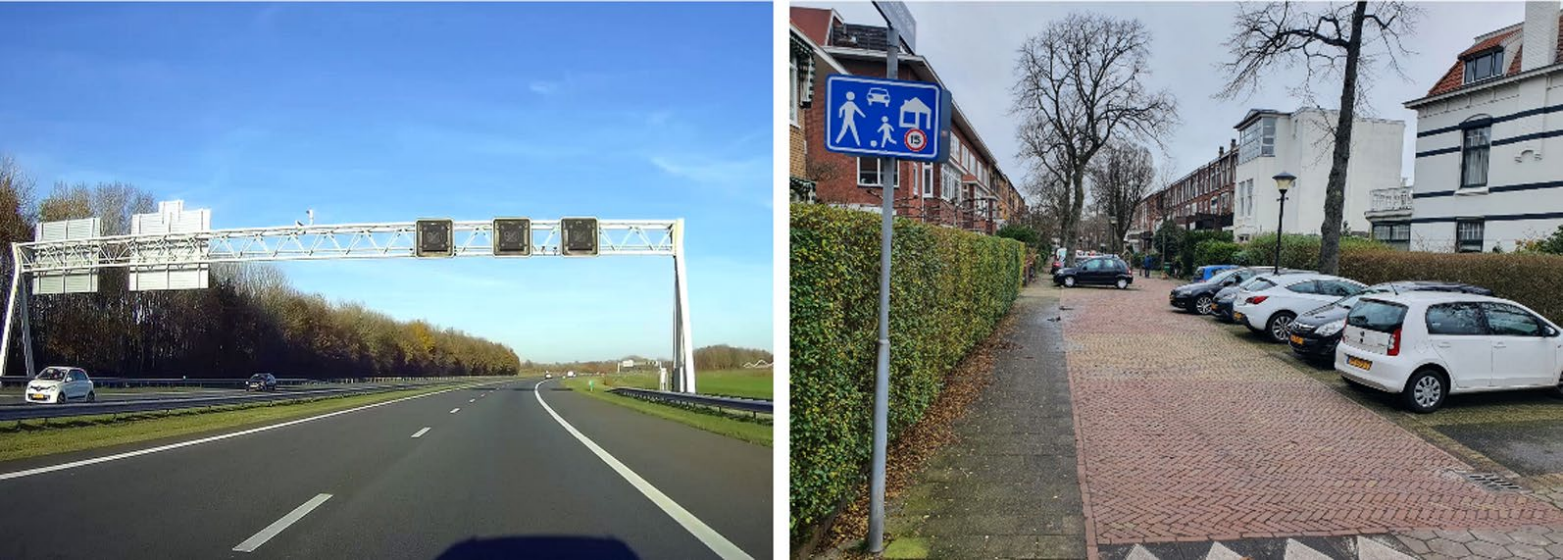
Two types of roads which can be considered to be self-explaining. On the left a typical Dutch motorway with defining features for fast driving such as wide lanes, smooth asphalt, an emergency lane, guard rails, overhead lane control signs, no sharp curves and a lot of preview. On the right, a typical Dutch woonerf (also known as “shared space”) designed for slow driving (maximum speed 15 km/h) with defining features such a physical barriers, shared and paced space and landscaping and street furniture

Also, roads that are called “woonerf” (originally developed in the Netherlands, sometimes referred to as “shared space”) are basically self-explaining. A woonerf is a street that is shared among pedestrians, bicyclists and motor vehicles; at all times, pedestrians and bicyclists have priority over cars. Usually, there is no clear division between bicyclists, pedestrians and car space; there is often street furniture (e.g., planters, street trees, benches) that are used as obstacles to slow down speed. In addition, there can be speedbumps and there are no delineations, markings or curbs. The whole design makes clear that motorists need to slow down and travel with caution. The design is self-explaining, and there is no need for traffic signs to indicate what is expected from road users entering these types of streets (see Fig. 4 for examples).

The roads that are the least self-explaining are rural roads that connect smaller cities and villages. In particular, these types of roads have different functions, show a large variation in appearance, have a mixture of road users (e.g., cars, cyclists, pedestrians), have various speed limits (basically from 50 to 100 km/h) and are unforgiving (i.e., when you make a mistake, the outcome may be fatal). Often the geometry and layout of these types of roads are not intentionally designed but instead are usually the result of some historical development (e.g., roads that used to be old horse trails connecting two cities become a road for cars and bikes). One could argue that rural roads are prime examples of not being “self-explaining.” The problem is that sometimes a rural road looks like a motorway which will induce wrong expectations. This may result in fast driving, not anticipating slow moving vehicles, crossing traffic, sharp curves or pedestrian crossing the street. As noted, there is a very large variation in their appearance. These roads can be narrow, bumpy and curvy or smooth straight and wide. Rural roads are prime examples of inadequately designed roads.

Given this analysis, it may not be surprising that, for example, in Europe (in 2018) 53% of road traffic fatalities occurred on rural roads, versus 38% in urban areas and only 9% on motorways/freeways (EU road safety statistics 2019). Undoubtedly, rural roads are the most dangerous road class in terms of the number of fatalities. As described by Weller (2010), the proportion of fatalities on rural roads is approximately 60% compared to 10% for motorways/freeways and 30% for inner urban roads (IRTAD 2007) with the proportion of fatalities on rural roads even rising over the last 25 years indicating that the other type of roads (motorways/freeways and inner urban roads) become safer (OECD 1999).

An exploratory study made a connection between self-explaining roads and situational awareness (SA) (Walker et al. 2013). SA is defined as understanding what is going on around you at any given time (e.g., Endsley 1995). The Walker et al. (2012) study showed that SA is highly dependent on the road type. It was argued that motorways/freeways are the most cognitively compatible road types and that incompatibilities grow when the roads are less deliberately designed for a particular purpose (for example, stretches of urban and rural road). This study suggests that the SA that drivers develop is linked to the appropriate driving behavior that is required on these roads.

#### Novice drivers

Novice drivers have less experience with the road environment and had therefore less time to learn the statistical regularities that exist in the road environment. As a consequence, novice drivers are less able to predict the location of critical objects and potential hazards than experience drivers. A study by Underwood et al. (1997) in which eye movement behaviour was measured is consistent with this notion. In this study, it was shown that experienced drivers have increased horizontal searches and decreased fixation durations relative to novices suggesting that experienced drivers scan the road environment for potential hazards much more than novice drivers do. A study by Crundall and Underwood (1998) provided also strong support for the notion that with driving experience, drivers learn to extract regularities in the environment. This study found that the visual scanning patterns of experienced drivers were adapted to specific road environments and situations, while novice drivers tended to use the same scanning patterns for all road types. Also, fixation durations for novice drivers were longer than that for experienced drivers. A study measuring eye movements while watching movies of the road environment showed that all drivers (novice and experienced) fixated objects that were salient in their environment; yet only experienced drivers fixated non-salient elements in the road environment that were crucial for the driving task (Borowsky et al. 2007) stressing the importance of experienced-based guidance of visual selection. Specifically, Borowsky et al. (2010) argued that the drivers’ awareness of potential hazards (or hazard perception) improves with driving experience as they showed that the eyes of experienced drivers are more likely to fixate those locations having potential risks.

The current analysis is consistent with Underwood (2007) who suggested that the efficiency of visual search strategies is one of the fundamental changes in skill that marks the transition from novice to experienced driver. It is likely that inexperience with the road environment and the inability to adapt scanning patterns that fit the road environment and road conditions, can explain why drivers with three years of experience had three times the number of accidents per year than drivers with 20 years of driving experience (Crundall et al. 1999).

#### Black spots

Even though there is no universally accepted definition of what a black spot is, the most common definition is that it is a road environment in which the registered number of accidents during a specific period is significantly higher than the number of accidents on a similar type of road or intersection. Typically, this is ranked on the basis of accident rate (for example, accidents per vehicle kilometers). In other words, these are road environment (intersections, curves, etc.) in which the probability of getting accident is much higher than on other similar types of road.

Even though there is little direct empirical evidence, it is likely that these locations that have a higher accident rate are locations which trigger the wrong expectations. We speculate that there are particular elements in the road environment that induces expectations that are not correct. If this occurs consistently, then such a location may be considered to be a “black spot.” For example, when approaching a roundabout outside the built-up area, an expectation maybe induced that bicyclists, if present, are likely to approach the roundabout from the left, while in fact there are situations in which they enter the roundabout from the right. If the approaching speed is relatively high (which may be induced from the environment approaching the roundabout) and there is some visual clutter, bicyclist approaching from the right is likely to be missed (see Räsänen and Summala 2000). Interestingly, enough this effect will be stronger among experienced drivers as they rely more on expectation induced scanning patterns.

#### Driving in a foreign country

Because what is learned depends on what one is exposed to, one can argue that those roads that are most often driven will affect road categorization most strongly, and therefore generate the strongest learned expectations. Hence, for each road user, his or her own environment, own city and country has the largest effect on the expectations developed. Therefore driving in one’s own country is often times much less stressful and takes much fewer resources than driving in a foreign country. When driving abroad, the learned pattern of scanning and anticipation of what will happen are often inappropriate and drivers may have trouble recognizing and categorizing the road environment. In fact, the experienced driver abroad may act more as a novice driver as the experienced driver cannot rely on his experience-based scanning. Interestingly, when driving abroad, it may be less dangerous when the road environment is completely different and does not resemble the road environment at home at all. Because everything is different (for example when driving for the first time on the left side in the UK), automatic patterns of visual scanning may not be automatically retrieved. The real danger is road environments that resemble the home situation but require completely different behavior compared to what the driver is used to.

There are very few studies that compared driving across different countries. In one study by Shinohara and Nishizaki (2017), Japanese drivers watched video clips of driving along a road in Japan and in San Francisco while eye movements were recorded. Interestingly, and consistent with our analysis this study showed that familiarity with the driving situation had a greater influence on experienced drivers than on novice drivers. For novices, there were basically no differences in eye movement patterns when driving along a Japanese (home) or US (foreign) route, probably because the lack of driving experience did not yet result in typical eye movement patterns for the familiar environment. For experienced drivers, however, there were differences between eye movements patterns with more saccades when driving along the unfamiliar foreign route relative to the familiar route.

## How to design traffic systems that are self-explaining

Traffic systems that they are self-explaining (SER) are systems that elicit safe and consistent behavior among the road users simply by its design. As outlined, road users should be able to categorize a road immediately and consistently as belonging to one or the other road type. In the Netherlands, the concept of self-explaining roads resulted in redefinition of three road types: Flow (Through), Distributor, and Access Road (see mobility and transport website of the European Commission). Roads with a flow function ensure an efficient throughput of (long distance) motorized traffic. There are typically a limited number of access and exit point. Typically all motorways/freeways, some through roads and some urban ring roads have such a flow function. There are normally no junctions with other traffic. Roads that have an area distributor function allow entering and leaving residential and recreational areas, industrial zones and rural settlements. The road should facilitate the flowing of traffic. There are junctions unregulated or regulated by traffic lights and/or roundabouts. Finally, roads with an access function make it possible to access to properties along street. There are junctions for traffic exchange and change of direction. The earlier discussed woonerf is a prime example of such a road.

### Principles of SER road design

#### General principles

- Roads should be *Easily Recognizable*: Roads that have the same function, the same speed profile, the same type of road users should look similar.
- Roads should be *Easy Distinguishable*: Roads of different categories should look differently. In other words, there should be clear differences in appearance and layout between roads that belong to different road categories.
- Roads should be *Easy Interpretable*: It should be clear from the design what the desired behavior should be on that route. The road characteristics should induce this type of behavior.

**Fig. 5:**
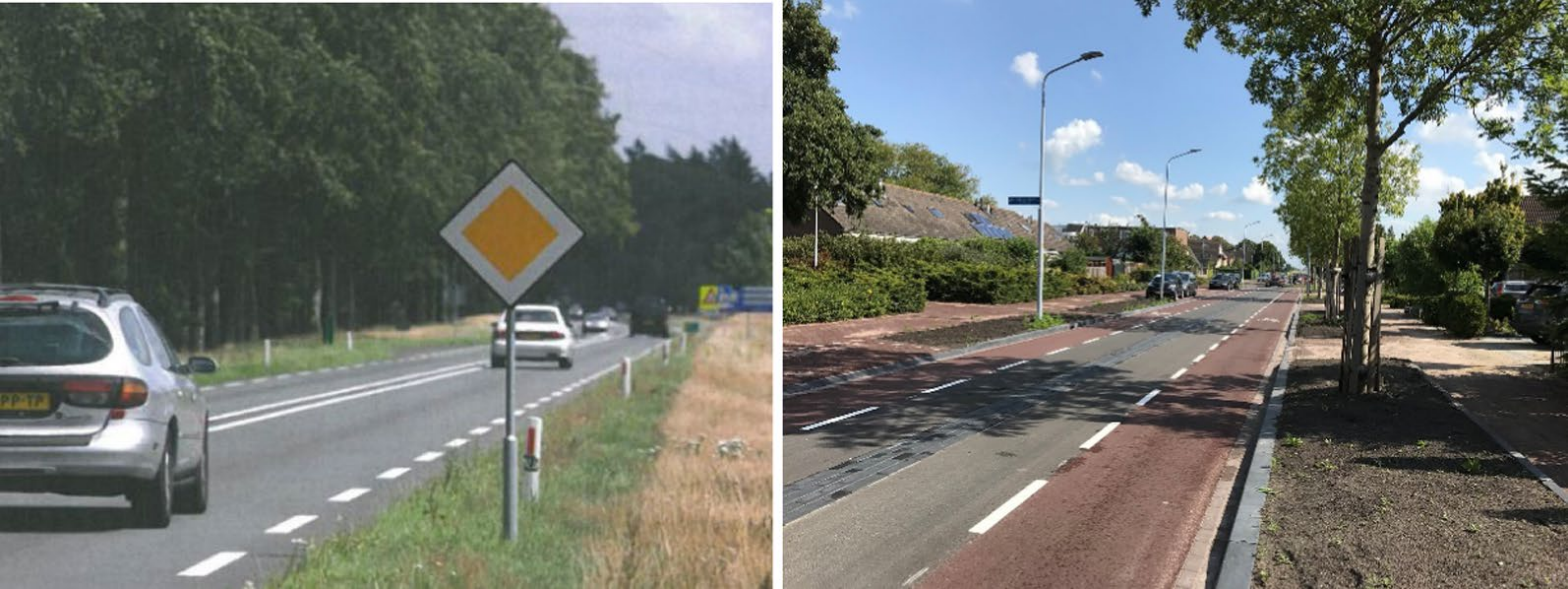
On the left a distributor road with double-line center markings and on the right an access road with red asphalt marking the bike path

To further illustrate this with some examples (see Fig. 5): If a road has a flow function, this should be easily recognizable by providing specific and unique visual features. For example in the Netherlands, typically these roads have a double white line as markings in the middle and broken lines along the road side. This marking makes the road type easy recognizable and immediately indicates that overtaking is not allowed. On these type of roads, one does not expect bicyclists while the marking of the access road (the red asphalt marking), immediately indicate that bicyclist can be present. Also, these types of access roads are narrow sometimes having obstacle (speed bumps) indicating low driving speeds and the possibility of oncoming traffic.

### Empirical evidence for SER design

#### Laboratory studies

Laboratory studies have shown that road users can categorize road types (Theeuwes and Diks 1995; Kaptein et al. 2002). As discussed earlier, Theeuwes and Diks’ (1995) key finding was that participants categorized roads that looked similar (had similar appearance) as being part of the same road category even though according to the official road classification were not part of the same classes of roads. Theeuwes and Diks (1995) also had participants estimate what they thought would be the appropriate driving speed on these types of roads. The results showed that the estimated speed fitted the subjective categorization much better than the official road classification (the categorization of the DOT). This implies that if road users categorize a road as being a motorway, they will drive a speed that is too high for that type of road. Similar findings were reported by Kaptein et al., (2002).

Weller et al. (2008) took the concept of self-explaining roads to investigate the subjective categorization of rural roads in Germany (see also Weller and Schlag 2007). As outlined earlier, rural roads are particularly dangerous with many more fatalities than the number of fatalities on motorways/freeways. Weller et al. (2008) developed a driver and driving behavior model which has strong resemblance to the ideas underlying SER. In this model, terms like “affordance and cues” (cf. Gibson 1968) are used which are the “category defining properties” of the SER concept. It is important that these categories are internally consistent, mutually exclusive and clearly distinguishable. Weller et al. (2008) used 25 pictures of existing rural roads and had participants categorize these. By means of a cluster analysis (e.g., Theeuwes and Diks 1995), the results indicated three different road clusters which were labeled as "Demand," "Comfort" and "Monotony." Importantly, as in Theeuwes and Diks (1995) the subjectively estimated appropriate speed in the road situation displayed on the picture fitted well with the subjective categorization, implying that the categorization is related to the type of behavior that people would display on these types of roads. This study confirms findings in showing that subjective road categorization is related to traffic behavior (e.g., Kaptein et al. 2002; Riemersma 1988; Theeuwes 1996, 2002).

In another laboratory study using a driving simulator, Kaptein and Claessens (1998) investigated the road design and how this affected speed choice. They investigated three different types of road categories outside the built-up area. One type of road was basically the standard way of road design as found in the Netherlands, one design was what was labeled as self-explaining roads (SER), and one was a combination of existing (current) road design with self-explaining elements. For the SER design, there was a strong overlap in characteristics within one road category and hardly any overlap between the road categories. Kaptein and Claessens (1998) showed that road users were better able to classify the SER designed as belonging to one or the other road type. For the basic standard and mixed designed, there was much less consistency in classifying these roads. The most important finding was that when participants were exposed to these different types of roads in the driving simulator, they choose more consistent and homogenous driving speeds for the SER design than for the standard regular road design indicating that the systematic and unequivocal categorization results in more systematic and homogenous driving behavior. Consistent with these findings, using series of photographs showing sections of two road categories, Stelling-Konczak et al. (2011) showed that road users discriminated between different types of rural roads when those roads were consistent with respect to the presence/absence of edge lines, physical separation between lanes and colored median treatments. Critically they also showed that participants identified the correct speed limits that were associated with these SER designed roads. Overall, these studies support the idea that road behavior is related to the appearance of the road and that road characteristics and traffic behavior are cognitively integrated by drivers into subjective categories.

#### Field/observational studies

Even though many road authorities adopted the SER principles, direct empirical evidence for its effectiveness is scarce because most studies lack appropriate control conditions. Several field and observational studies using some of the SER principles have been conducted. In a study by Herrstedt (2006) several roads were treated with SER measures and the speed driven before and after the treatment were measured. Even though it was mentioned that at some roads, drivers behave according to the measures taken, it was not clear from this study as to whether the actual driving speeds were affected by the measures since driving speeds were not reported. The impact of new SER road markings on speed driven on low volume German roads was studied by Richter and Zierke (2009). They reported that the speed dropped by around 10% after new marking was painted. Also, the speed profile was more homogenous which increases traffic safety (see also Kaptein et al. 2002).

Perhaps the most direct empirical evidence for the effects of SER principles is from a field study of Charlton et al. (2010) which was carried out in New Zealand. This study was designed to identify and develop guidelines that would enable the development of speed management using the self-explaining approach. The approach was formulated by the Ministry of Transport of New Zealand as follows: “*The emphasis is not just on speed limit enforcement, it includes perceptual measures that influence the speed that a driver feels is appropriate for the section of road upon which they are driving–in effect the ‘self-explaining road*'” Charlton and Baas 2006; p. 7). In this study, some roads received a SER treatment such that there were maximum differences between the different road categories while other roads served as matched control roads. The SER design for local roads entailed landscaping and the creation of community islands to limit forward visibility, and the removal of road markings to create a visually distinct road environment. Roads that were categorized as collectors (“distributor roads”) had increased delineation, addition of cycle lanes and specific design solutions for pedestrians. After the implementation of these measures, speed data were collected for 3 months. The results were quite dramatic as there was a large and significant reduction in speed driven on these treated roads compared to matched control roads (a reduction of about 15 km/h). In addition, the variance in speed driven on these roads was reduced significantly suggesting more homogeneous behavior. Overall, the project in New Zealand was very successful and demonstrated that SER is very effective. It was argued that a clear multilevel road hierarchy was established with each level having a distinct “look and feel” and discriminability of different speed profiles. Note that also rating of local residents regarding the appearance of these roads showed significantly more positive ratings following installation of the SER treatments.

In a follow-up study, Mackie et al. (2013) recorded videos over nine separate days at nine different locations both before and after SER construction. Following SER construction, local roads became more user-friendly with less through traffic and a higher proportion of pedestrians. It was argued that implementing SER constructions on local roads made these roads more what they are supposed to be: a more informal/low speed local road environment in which pedestrians were less constrained. This effect was not found on collector roads (“distributor roads”) which fits with the idea that the road design should be consistent with the type of behavior that is required.

A recent study by Yao et al. (2020) investigated the “credible” speed limits in the UK, which is defined as the speed limit that is accepted by most drivers without the need of enforcement. They concluded consistent with the SER principles that speed limit credibility depended very much on the characteristics of the road layout. Specifically, rural motorways gave the most uniform driving speed and were considered as excellent examples of SER. Urban motorways, however, were considered to be not self-explaining because most drivers considered the speed limit on these roads (40 mph) as not credible. In other words, the road environment suggested much higher speeds than was allowed on these types of roads.

#### Other developments

Over the last 25 years, many countries adopted the SER principles as the basis for road design. In the Netherlands, the principle of SER became one of the main guidelines of road safety policy (Aarts and van Schagen 2006; Aarts et al. 2005; Kraay 2002; Wegman and Aarts 2006; Wegman 1995). The idea of SER was also applied to improve cycling safety in the Netherlands (Schepers et al. 2014). Germany embraced SER principles into their national guidelines for rural roads (Matena and Weber 2009; Richter and Zierke 2009; Weber and Hartkopf 2005). In the UK, it was recognized that traditional methods of reducing speed were ineffective and that total road environment should be enhanced using self-explaining road design (Shaw and Mayhew 2000). Similar ideas in the UK were put forward by Elliott et al. (2003) and Kennedy et al. (2005). In 2017, the Czech Republic used the SER principles to increase the level of safety on their national roads (Ambros and Valentová 2013; Ambros et al. 2019). In 2019, Belarus used SER principles for developing a new road traffic safety concept (Kapsky et al. 2019). In Hungary, the cross-sectional designs of rural roads were analyzed determining the extent to which the roads were self-explaining (Torok 2013). A study from Israel showed that changing shoulder width, recovery-zone width or junction density may be applied for promoting the SER concept and likely affects travel speeds (Gitelman et al. 2016).

Baas and Charlton (2005) introduced the SER principle in New Zealand. Australia launched the "Safe System Infrastructure" initiative (Turner et al. 2009, p7), in which they describe explicitly “*a self-explaining road is a term from the Netherlands which describes a road which is designed in such a way that drivers will automatically understand what is required of them, including speed choice*” (see also Fildes and Lee 1993). In China, the principles of SER were applied in a simulation study determining which elements in the road environment would determine driving speed (Qin et al. 2020). In India, which has a very high fatality rate, the need for better road design and geometric standards is stated including the idea of using SER principles (Tiwari 2015). In 2014, the roads of the Sultanate of Oman (which has one of the highest road fatalities worldwide) were evaluated with respect to the extent to which they can be considered as self-explaining. This study showed that the roads of the Sultanate of Oman were not designed according to the SER principles (Plankermann 2014). A group of American highway engineers visited Europe in 2001 to learn more about potentially transferable practices including SER (Brewer et al. 2009). Regardless of this effort, the concept did not catch on in the US to the same extent as in the rest of the world.

## Summary and conclusions

The present paper describes in detail the theoretical basis for the idea of self-explaining roads and why this may have such a large effect on human behavior. The notion is firmly rooted in the theoretical framework of statistical learning, subjective categorization and the associated expectations. Worldwide this concept has been embraced by road authorities, politicians and engineers as an approach for redesigning the road environment. The paper describes some of its successful implementations and recent developments worldwide.

## Data Availability

Review, no data, no material.
